# Assessment of clients satisfaction with outpatient services at Yekatit 12 Hospital Medical College, Addis Ababa, Ethiopia

**DOI:** 10.1186/s13104-018-3603-3

**Published:** 2018-07-27

**Authors:** Tirhas T. Berehe, Getabalew E. Bekele, Yimer S. Yimer, Taye Z. Lozza

**Affiliations:** 1 Department of Public Health, College of Medicine, Yekatit 12 Hospitals, Addis Ababa, Ethiopia; 2 Department of Preventive Medicine, School of Public Health, Addis Ababa University, Addis Ababa, Ethiopia

**Keywords:** Patient satisfaction, Outpatient, Yekatit 12 Hospital Medical College

## Abstract

**Objective:**

This study aimed at assessing clients’ satisfaction and associated factors among adults. A cross sectional facility based study was conducted on 420 clients of Yekatit 12 Hospital Medical College from 1 June 2016 to 1 July 2016. Data was entered, cleaned, and analyzed using SPSS statistical package. Data was analyzed using a multivariable logistic regression model to find out the most significant predictors for clients satisfaction with outpatient services at Yekatit 12 Hospital Medical College.

**Results:**

This study showed that the overall clients’ satisfaction level towards out-patient health service at Yekatit 12 Hospital Medical College was 47% at 95% CI (42.5, 51.7%). The most frequently identified problems were: lack of clean toilet in nearby the waiting areas, lack of waiting area particularly at pharmacy, inadequate furniture like chair, lack of adequate drugs and supplies, lack of privacy at the examination room, lack of direction signs, and poor communication between clients and health service providers. In conclusion the overall satisfaction level of the patients is low, so this demands the Hospital to take further action on the identified problems to improve the services delivered to the patients.

**Electronic supplementary material:**

The online version of this article (10.1186/s13104-018-3603-3) contains supplementary material, which is available to authorized users.

## Introduction

Increasing awareness on healthcare service brings increased utilization of healthcare. Evidence of variations in satisfaction with clinical practices in many health service institutions in Ethiopia have raised interest in measuring and improving healthcare service to enhance customer satisfaction [1]. According to the Donabedian model [2], three interrelated key domains namely structure, process, and outcome are important for identifying clients’ satisfaction which is by far an important indicator of service quality [2, 3]. Therefore, client satisfaction has been found to be the most useful tool for getting clients’ views on how to provide health care service. Generally, clients are the best source of information on both quality and quantity of health care services. Moreover, clients’ views provide useful information that can be used in planning and providing improved health care service [4].

But client perception about care is neglected area by many health managers and clinicians in developing countries [5]. Taking this into consideration, the study aimed at determining the level of clients’ satisfaction towards out-patient service at Yekatit 12 Hospital Medical College Addis Ababa Ethiopia. The result from this study identifies the problems, which can be used to improving health service delivery through informed strategies.

## Main text

### Methods

This study was conducted at Yekatit 12 Hospital Medical College (Y12HMC) outpatient department from 1 June 2016 to 1 July 2016. The specialized hospital is found in Addis Ababa, the capital city of Ethiopia. It is serving more than 5 million people in the catchment area (see Additional file 1). Quantitative approaches were applied in the study. All clients visiting the hospital for outpatient health services from 1 June 2016 to 1 July 2016 were the source population and clients coming to the outpatient departments during the study period were selected using the systematic random sampling technique was the study population. The exclusion criteria for this study were; the very seriously ill clients who did not have somebody to accompany them because of the difficulty of interviewing such cases (getting the consent, lack of tolerance of the pain or illness and etc.), and clients coming for the second time during the study period and children who are under 15 who are alone. Quantitative approaches were applied in the study. The sample size was 423 which were determined using one population proportion formula. A systematic random sampling method using patients’ registration number as a sampling frame was employed to select respondents (see Additional file 2).

To collect relevant data, structured questionnaire was developed through reviewing related literature for the study [6–9]. Then, the validity of the questionnaire was tested after translating in to Amharic language. The lexical equivalence of questionnaire was examined after back translation. Content validity was established by a panel of experts from public health, community medicine, pharmacy, laboratory technician, nursing, epidemiology, biostatistics, and health education. The final version of the questionnaire had a good indicator for reliability (as indicated by Alpha Cronbach test value of 0.809) (see Additional file 3).

The dependent variable of this study was overall clients’ satisfaction. Out-patient service satisfaction is defined as meeting the perceived needs and the expectations of the client in relation to factors related to the health care provider and amenities. In order to measure the overall clients satisfaction, respondents were asked eight questions with likert five point scale (e.g. I am satisfied with overall cleanliness of the hospital compound) to describe their level of agreement in a five scale response. The 5-point liker scale response options, scored from 1 to 5, were 1 = strongly disagree, 2 = disagree, 3 = neutral, 4 = agree, and 5 = strongly agree. Subscale scores were obtained by summing item scores and dividing by the total number of items. If it was above or equal to the average, it would be indicative for good satisfaction.

Data were collected by literate and trained data collectors who were health professionals who are not working in the study site for 30 days with an average 14 exit interviews per day conducted in six confidential rooms using a structured and pre-tested questionnaire (see Additional file 3).

Binary logistic regression analysis was carried out to see the association between each independent variable with the outcome variable. Then, variables that showed significant associations at P-value of 0.2 was included in a single model and multiple logistic regressions were performed to identify the most significant predictors. The 95% CI and a P-value of 0.05 were used to assess the degree of statistical significance.

### Results

#### Socio-demographic characteristics of the participants

Among the total samples (n = 423) 420 adult patients’ were interviewed, giving a response rate of 99.3%.

Out of the total study subjects, 273 (65.5%) were females. Two hundred fifteen (51.2%) of the respondents were between the age group of 35–54 years. The mean age of the respondents was 39.8+ (SD) (11.4) years, and 302 (71.9%) of them were married. Concerning the religion and ethnicity of the respondents, the majority 341 (81.2%) were orthodox and 235 (56%) were Amhara respectively (Table 1).

**Table 1:** Socio-demographic characteristics of the study participant at Yekatit 12 Hospital Medical College, Addis Ababa June 2016

S/N	Socio-demographic variables	Frequency	Percent
1	*Age*		
	≤ 34	143	34
	35–54	215	51.2
	≥ 55	62	14.8
2	*Sex*		
	Male	145	34.5
	Female	273	65.5
3	*Marital status*		
	Married	302	71.9
	Single	81	19.3
	Others (divorced, widowed and cohabited)	37	8.8
4	*Ethnicity*		
	Amhara	235	56
	Oromo	83	19.7
	Guragie	49	11.7
	Tigray	23	5.5
	Others (Wolita, Hadiya, kenbata, yem)	30	7.1
5	*Religion*		
	Orthodox	341	81.2
	Muslim	44	10.5
	Protestant	24	5.7
	Catholic	14	3.3
4	*Educational status*		
	Illiterate	16	3.8
	Read and write	17	4
	Grade 1–6	92	21.9
	Grade 7–12	151	36
	Diploma and above	144	34.3
5	*Occupation*		
	House wife	161	38.3
	Merchant	82	19.5
	Government employee	58	13.8
	No job	55	13.1
	Student	33	7.9
	Retire	20	4.8
	Farmer	11	2.6
6	*Income*		
	< 1500	247	58.8
	≥ 1500	173	41.2
7	*Payment status (Did you pay money for the services?)*		
	Paying	271	64.5
	Free	124	29.5
	Credit	25	6
8	*Did you consider the outpatient visit is too expensive*		
	Yes	164	55.4
	No	132	44.6
9	*Type of visit*		
	Repeat	374	89
	New	89	11

#### Outpatient service related characteristics of the participants

One hundred sixty three (38.6%) of the participants were satisfied with the location of each service area in the hospital. Respondents were asked about their level of satisfaction regarding the availability of enough waiting chairs and the spaciousness, brightness and airiness of the waiting room, only 80 (19%) were satisfied. Concerning the toilet room out of the total 420 respondents only 355 (84.5%) clients visited the toilet, of those only 88 (21%) were satisfied with cleanliness and accessibility of the toilet.

One hundred thirty nine (33.1%) of the participants were satisfied with the availability of drugs and supplies. One hundred thirty one (31.2%) of the respondents were satisfied with the cares given to assure privacy at all service delivery points. Concerning the interaction with the health care providers and supportive staffs, about 163 (38.8%) of the participants were satisfied with interaction they had with doctors/health officers at the outpatient department.

Concerning interaction with nurses, 182 (43.3%) of the respondents were satisfied. Furthermore, from the total 358 participants who received laboratory service, 120 (33.5%) of them were satisfied with the interaction of the laboratory staffs respectively. One hundred sixty six (39.5%), and 71 (19.9%) of the respondents were satisfied with the communication of the pharmacy staffs and card registration staffs. Generally, this study revealed that 197 (47%) of the respondents were satisfied with the overall service provided in the outpatient department with median value 33, mean value of 32.2 and standard deviation of ±4.1 (Table 2).

**Table 2:** Outpatient service related characteristics of the study participant at Yekatit 12 Hospital Medical College, Addis Ababa June 2016

S/n	Variables	Frequency	Percent
1	*Sign and direction indicators were available to ease ways*		
	Yes	163	38.6
	No	257	61.2
2	*Over all comfort of waiting area of services*		
	Yes	80	19
	No	340	81
3	*Have got all ordered of prescribed drugs*		
	Yes	139	33.1
	No	281	66.9
4	*Accessibility of the toilet*		
	Yes	88	21
	No	340	81
4	*Cleanliness of the toilet*		
	Yes	88	21
	No	332	79
5	*Privacy maintained*		
	Yes	131	33.2
	No	289	68.8
6	*Interaction with doctors*		
	Good	163	38.8
	Bad	257	61.2
7	*Interaction with registration card*		
	Good	71	16.9
	Bad	349	83.1
8	*Interaction with nurses*		
	Good	182	43.3
	Bad	238	56.7
9	*Interaction with pharmacy*		
	Good	166	39.5
	Bad	254	60.5
10	*Interaction with laboratory*		
	Good	120	33.5
	Bad	238	66.5
11	*Overall level of satisfaction*		
	Satisfied	197	47
	Dissatisfied	223	53

#### Association between the level of satisfaction and selected variables

Binary logistic regression was conducted using bi-variate analysis for socio-demographic variables, and outpatient service related variables. Those variables with P-values less than 0.2 at bi-variate analysis were included in multivariable analysis. In multivariable analysis, factors that remained statistically significant with patient satisfaction were availability of direction indicators (AOR 1.2, 95% CI 1.01–2.43), privacy respected during consultation (AOR 2.9, 95% CI 2.33, 3.76), cleanliness of the toilet (AOR 0.79, 95% CI 0.43, 0.99), availability of prescribed drugs in hospital pharmacy (AOR 1.9, 95% CI 1.12–2.88), and interaction with registration room workers (AOR 5.3, 95% CI 2.68–5.39 (Table 3).

**Table 3:** Association patients level of satisfaction with the selected variables in outpatient department of Yekatit 12 hospital medical colleges Addis Ababa, June 2016

Variables	Level of satisfaction		Crude OR (95% CI)	Adjusted OR (95% CI)
	Dissatisfied	Satisfied		
Sex				
Male	84	139	0.8 (0.52, 1.17)*	1.2 (0.78, 1.83)
Female	63	134	1	1
Age				
≤ 34	77	66	0.9 (0.50, 1.66)	0.2 (0.07, 0.56)
35–54	114	101	0.9 (0.54, 1.66)	1.2 (0.45, 3.10)
≥ 55	32	30	1	1
Family monthly income				
< 1500	205	144	0.9 (0.58, 1.27)	
≥ 1500	88	85	1	
Payment status				
Paying	144	127	1.9 (0.78, 4.49)*	0.4 (0.18, 1.11)
Free	62	62	2.1 (0.85, 5.39)*	0.4 (0.14, 1.01)
Credit	17	8	1	1
Sign and direction indicators were available to ease ways				
Yes	106	57	0.7 (0.11, 0.96)*	1.2 (1.01, 2.43)**
No	202	55	1	
Privacy maintained during consultation				
Yes	31	100	2.6 (2.11, 3.32)*	2.9 (2.33, 3.76)**
No	201	88	1	
Cleanliness of the toilet				
Yes	39	49	0.59 (0.37, 0.97)	0.79 (0.43, 0.99)**
No	300	32	1	1
Have got all ordered of prescribed drugs				
Yes	49	90	1.3 (1.12, 1.89)*	1.9 (1.12, 2.88)**
No	230	52	1	1
Interaction with Dr./HO				
Bad	74	183	1.6 (1.02, 3.93)*	2.8 (1.74, 4.96)
Good	55	108	1	1
Interaction with nurse				
Bad	145	93	0.5 (0.32, 0.71)*	1.9 (1.16, 3.06)
Good	78	104	1	1
Interaction with lab				
Bad interaction	106	87	0.8 (0.52, 1.20)*	7 (2.71, 18.11)
Good interaction	81	84	1	1
Interaction with pharmacy				
Bad	201	53	0.8 (0.51, 1.11)*	0.6 (0.34, 0.95)
Good	81	85	1	1
Interaction with registration				
Bad	205	144	0.2 (0.13, 0.42)*	5.3 (2.68, 5.39)**
Good	18	53	1	1

NB * statistically significant as the P value is < 0.2 for crude OR

** Statistically significant as P value is < 0.05 for adjusted OR

### Discussion

This study showed that the overall satisfaction level of the outpatient services provided at Yekatit 12 Hospital Medical College (Y12HMC) was 47% at 95% CI (42.5, 51.7%).

This result is similar with the study done in Molango Hospital Uganda and Wolita Sodo University Teaching Hospital [6, 10]. The present study suggested that overall satisfaction level report is low compared to the report of the studies conducted in, Bahirdar Felege Hiwot Referral Hospital, and Hawassa University Teaching Hospital, which showed 52.8 and 80.1% respectively [3, 10, 11]. Concerning the privacy, almost 88% of the participants did not have private consultation.

Furthermore, the regression analysis revealed that private consultation increased satisfaction by 97.1% at 95% CI 2.9 (2.33, 3.76). This is similar with a study conducted in the University of Gondar Referral and Teaching Hospital, Ethiopia [12]. However, this is extremely higher compared with other studies conducted in Felegehiwot Referral Hospital, North West Ethiopia (26%) [11], Hawasa University Teaching Hospital 18.6% [3], Wolaita-Sodo University Teaching Hospital, Southern Ethiopia 20% [10]. This difference might be due to the difference in the study areas.

According to the study conducted in India, easy accessibility and a good signage system for the OPD services provide a good image for the hospital [7]. However the researchers revealed that there was a statistically significant association between the absence and presence of direction sign against client satisfaction. The presence of signing direction increased satisfaction by 98.8%. This finding was consistent with the previous study conducted in Bahirdar Ethiopia in which 74% of the participants were dissatisfied due to the absence of signing direction [11].

Pertaining to the cleanliness of the toilet, availability of clean toilet increased patient satisfaction by 79% at 95% CI 0.79 (0.43, 0.99). This is similar with the study done in Wolita-Sodo University Teaching Hospital [10], and the Felege-Hiwot Hospital Bahirdar, Ethiopia in which 62% of the participants were dissatisfied due to luck of clean toilet [11].

Regarding the availability of drugs and supplies in the hospital, there was a significant association between the availability of drugs and overall level of satisfaction (AOR 1.9, 95% CI 1.12–2.88). This is similar with the study conducted at private wing of Felegehiwot Hospital, Bahirdar and Hawassa University Teaching Hospital [3, 11].

### Conclusion

The overall client satisfaction in Yekatit 12 Hospital Medical College was 47% at 95% CI (42.5, 51.7%) which was low as compared to different studies in Ethiopia and outside Ethiopia. High proportions of patients were dissatisfied with patient satisfaction measuring items. In order to gain better views of the field and produce more meaningful result, further study should be conducted with broader scope using comparative studies.

## Limitation

The finding of this study is interpreted in light of several limitations. The cross-sectional design gives only a snap shot of events. Social desirability bias is also likely in this study as the respondents were interviewed in the compound of the health facility.

## Additional files

**Additional file 1.** Population.

**Additional file 2.** Sample size determination and sampling procedure.

**Additional file 3.** Data collection.

## Abbreviation

Y12HMC: Yekatit 12 Hospital Medical College
