# Supplementary material for: Assessment of clients satisfaction with outpatient services at Yekatit 12 Hospital Medical College, Addis Ababa, Ethiopia
Source: BMC Res Notes. 2018 Jul 27;11:507. doi: 10.1186/s13104-018-3603-3 (PMC6063000; doi:10.1186/s13104-018-3603-3)
Supplement: Supplementary file 1 — Additional file 1. Population. [file 13104_2018_3603_MOESM1_ESM.docx]

**File name**: Additional file 1:

**Title of the data**: Population

**Description of data**:The study was conducted Yekatit 12 specialized Hospital Medical College (Y12HMC) in OPD regular working hours from June 1-Julay 1,2016. The specialized hospital is found in Addis Ababa the capital city of Ethiopia. The hospital is serving more than 5 million people in the catchment area in all six major departments and other units since 1923. During the study period, Yekatit 12 hospital medical use to attend 200 to 250 patients per day through six units at the OPD
