# Supplementary material for: Assessment of clients satisfaction with outpatient services at Yekatit 12 Hospital Medical College, Addis Ababa, Ethiopia
Source: BMC Res Notes. 2018 Jul 27;11:507. doi: 10.1186/s13104-018-3603-3 (PMC6063000; doi:10.1186/s13104-018-3603-3)
Supplement: Supplementary file 2 — Additional file 2. Sample size determination and sampling procedure. [file 13104_2018_3603_MOESM2_ESM.docx]

**File name**: Additional file 2:

**Title of the data:** Sample size determination and sampling procedure

Total of 423 Sample size was determined based on Proportion of patients satisfaction without patient services hospital care services to be 80.1% %, according to a study done in Hawasa University teaching Hospital Southern Ethiopia (3) , Expected margin of error (d) of 0.04 and with 95% confidence level (Za/2) and 10% contingency for non-response. Allocation of samples into outpatient departments was done proportionately. The proportionate allocation was done by considering the average number of patient flow of the six departments in the same month of the preceding year (2015) and the month prior to the actual data collection period (2016). A systematic random sampling method which used patients’ registration book as a sampling frame was employed to select respondents. The sampling interval (k = 15) was calculated by dividing the source population to the total sample size (423) and this interval was used in all OPD to select study subjects. The first client was selected by simple random sampling among the first six OPD service users in the sample frame.

The questionnaire was pretested on clients of Yekatit12 Hospital Medical College one week prior to the actual data collection period. Pretesting was done aiming to check the sequence of questions, and comprehensiveness of the questions among the participants. Based on the feedback found from the pretest the data collection tool was slightly modified. The reliability and validity of the tool was also checked again using Cronbach’s Alpha test and was found to be acceptable (0.809).
