# Supplementary material for: Assessment of clients satisfaction with outpatient services at Yekatit 12 Hospital Medical College, Addis Ababa, Ethiopia
Source: BMC Res Notes. 2018 Jul 27;11:507. doi: 10.1186/s13104-018-3603-3 (PMC6063000; doi:10.1186/s13104-018-3603-3)
Supplement: Supplementary file 3 — Additional file 3. Data collection. [file 13104_2018_3603_MOESM3_ESM.docx]

**File name**: Additional file 3

**Title of the data: Data collection**

**Description of data:** Data collection Exit interviews of patients were conducted in six confidential rooms using a structured and pre-tested questionnaire. Data were collected by literate and trained data collectors who were health professionals (nurses) from the nearby health centers or district health offices in order to minimize interviewer bias.
